# SEOM clinical guidelines for the management of germ cell testicular cancer (2016)

**DOI:** 10.1007/s12094-016-1566-1

**Published:** 2016-11-04

**Authors:** J. Aparicio, J. Terrasa, I. Durán, J. R. Germà-Lluch, R. Gironés, E. González-Billalabeitia, J. Gumà, P. Maroto, A. Pinto, X. García-del-Muro

**Affiliations:** 1 Servicio de Oncología Médica, Hospital Universitario y Politécnico La Fe, Av. Abril Martorell 106, 46026 Valencia, Spain; 2 Hospital Universitario Son Espases, Palma de Mallorca, Spain; 3 Instituto de Biomedicina de Sevilla, IBiS/Hospital Universitario Virgen del Rocío/CSIC/Universidad de Sevilla, Sevilla, Spain; 4 Institut Català d’Oncologia, ICO-IDIBELL L’Hospitalet, Barcelona, Spain; 5 Hospital Lluis Alcanyis, Xátiva, Spain; 6 Hospital Universitario Morales Meseguer-IMIB, Universidad Católica de Murcia-UCAM, Murcia, Spain; 7 Hospital Universitario Sant Joan de Reus, URV, IISPV, Reus, Spain; 8 Hospital de Sant Pau, Barcelona, Spain; 9 Hospital Universitario La Paz, Madrid, Spain

**Keywords:** Testicular cancer, Germ-cell tumor, Chemotherapy, Surgery

## Abstract

Testicular cancer represents the most common malignancy in males aged 15–34 years and is considered a model of curable neoplasm. Maintaining success, reducing treatment burden, and focusing on survivorship are then key objectives. Inguinal orchiectomy is the first recommended maneuver that has both diagnostic and therapeutic aims. Most patients are diagnosed with stage I disease (confined to the testicle). Close surveillance and selective, short-course adjuvant chemotherapy are accepted alternatives for these cases. In patients with more advanced disease (stages II and III), 3–4 courses of cisplatin-based chemotherapy (according to IGCCCG risk classification) followed by the judicious surgical removal of residual masses represent the cornerstone of therapy. Poor-risk patients and those failing a first-line therapy should be referred to specialized tertiary centers. Paclitaxel-based conventional chemotherapy and high-dose chemotherapy plus autologous hematopoietic support can cure a proportion of patients with relapsing or refractory disease.

## Introduction

To develop updated, accurate clinical guidelines , the Spanish Society of Medical Oncology (SEOM) and the Spanish Germ Cell Cancer Group (SGCCG) invited ten experts based on major scientific contribution in the field of germ-cell testicular cancer (GCTC). The purpose of this paper was to define current ‘‘state-of-the-art’’ in the treatment of this malignancy using the methodology of evidence-based medicine. The available medical literature was reviewed according to main topics of disease management, and classified by scientific levels of evidence and grades of clinical recommendation according to the Infectious Diseases Society of America grading system [1]. The resulting text was reviewed, discussed, and approved by all authors.

## Epidemiology

In 2012, around 55,000 new cases of germ cell testicular cancer were diagnosed worldwide. This represents about 1.5% of all cancer diagnosis globally [2]. Despite this overall low occurrence, GCTC is the most frequent malignancy in males aged 15–34 years and, therefore, represents a substantial medical challenge. Incidence has increased over the last decades [3] due to non-completely understood reasons and differs considerably across world regions. Age-standardized incidence rates (ASR) range from 1–2/10^5^ in Africa and most Asia to 9–12/10^5^ in Scandinavia. Spain is considered an intermediate ASR country with 3.5 cases per 10^5^ making a total of around 800 new GCTC patients diagnosed every year [2]. Initiation of pathogenesis is believed to occur in most cases intrauterine where primordial germ cells would escape normal differentiation to become germ cell neoplasia in situ (GCNIS) that during puberty would gain invasive capacity [4]. The exact aetiology of GCTC is not clear. The most solid epidemiological risk factors are cryptorchidism, with 2–18 fold increased risk and a previous history of GCTC, with around 5% of patients developing a second tumor in the remaining testicle.

## Histopathology

Based on the most recent histological taxonomy of GCTC according to the World Health Organization classification (2016 version), testicular germ cell tumors are now divided into two different groups: those derived from GCNIS and those unrelated to GCNIS [5]. The former comprises a number of histological variants that have similar epidemiologic links and happen in a background of disturbed testicular development with typical morphologic features and amplification of genetic material through an isochromosome 12p. These tumors represent progression from GCNIS and consist of pure forms of seminomas and non-seminomas along with mixed forms. The germ cell cancers unrelated to GCNIS include the spermatocytic tumor (previously named spermatocytic seminoma) and some pre-puberal histological variants (Table 1).

**Table 1:** Histological classification of testicular germ cell tumors according to World Health Organization classification 2016 [5]

**Germ cell tumors derived from germ cell neoplasia in situ**
Non-invasive germ cell neoplasia
Germ cell neoplasia in situ
Specific forms of intratubular germ cell neoplasia
Tumors of a single histological type (pure forms)
Seminoma
Seminoma with syncytiotrophoblast cells
Non-seminomatous germ cell tumors
Embryonal carcinoma
Yolk sac tumor, postpubertal-type
Trophoblastic tumors
Choriocarcinoma
Non-choriocarcinomatous trophoblastic tumors
Placental site trophoblastic tumor
Epithelioid trophoblastic tumor
Cystic trophoblastic tumor
Teratoma, postpubertal-type
Teratoma with somatic-type malignancy
Non-seminomatous germ cell tumors of more than one
Histological type
Mixed germ cell tumors
Germ cell tumors of unknown type
Regressed germ cell tumors
**Germ cell tumors unrelated to germ cell neoplasia in situ**
Spermatocytic tumor
Teratoma, prepubertal-type
Dermoid cyst
Epidermoid cyst
Well-differentiated neuroendocrine tumor (monodermal teratoma)
Mixed teratoma and yolk sac tumor, prepubertal-type
Yolk sac tumor, prepubertal-type

Reproduced with permission from Moch H, Humphrey PA, Ulbright TM, Reuter VE. World Health Organization Classification of Tumors of the Urinary System and Male Genital Organs. IARC, Lyon, 2016

## Diagnosis and staging

In patients with a testicular mass, bilateral testicular ultrasound serves to confirm its presence and to explore the contralateral testis. The minimum mandatory tests are (recommendation grade A): physical examination, complete blood count, serum creatinine, electrolytes and liver enzymes, pre- and post-orchiectomy serum tumor markers [alpha-fetoprotein (AFP), beta-human chorionic gonadotropin (BHCG) and lactate dehydrogenase (LDH)], chest (mandatory in non-seminoma), abdomen, and pelvic CT scan. A discussion with the patient about sperm banking is recommended.

Other tests: bone scan or spinal MRI is recommended in case of symptoms, brain imaging (CT scan or MRI) in the event of neurologic manifestations, and for patients with extensive metastatic disease and/or very high BHCG values. There is no evidence to support the use of fluorodeoxyglucose-PET (FDG-PET) in the staging of testis cancer.

Inguinal orchiectomy is both diagnostic (histology and staging) and therapeutic. Rarely, when a patient presents with rapidly increasing BHCG, symptoms related to disseminated disease and a testicular mass, chemotherapy can be initiated immediately without waiting for a histological diagnosis.

The TNM staging system is based on post-orchiectomy findings according to the American Joint Committee on Cancer (AJCC), and AFP, BHCG, and LDH values [6] (Table 2). Serum tumor markers post-orchiectomy and before the start of chemotherapy are important to classify the patient according to the International Germ Cell Cancer Collaborative Group (IGCCCG) classification [7] (Table 3).

**Table 2:** TNM classification for testicular cancer (UICC, 2009, 7th ed.) [6]

*Primary tumor* (*T*)	
pTx	Primary tumor cannot be assessed (is used if no radical orchiectomy has been performed)
pT0	No evidence of primary tumor
pTis	Intratubular germ cell neoplasia (testicular intraepithelial neoplasia)
pT1	Tumor limited to testis and epididymis without vascular/lymphatic invasion: tumor may invade tunica albuginea but not tunica vaginalis
pT2	Tumor limited to testis and epididymis with vascular/lymphatic invasion, or tumor extending through tunica albuginea with involvement of tunica vaginalis
pT3	Tumor invades spermatic cord with or without vascular/lymphatic invasion
pT4	Tumor invades scrotum with or without vascular/lymphatic invasion
*Regional lymph nodes* (*N or pN*)	
pNx	Regional lymph nodes cannot be assessed
pN0	No regional lymph nodes metástasis
pN1	Metastasis with a lymph node mass 2 cm or less in greatest dimension and 5 or fewer positive nodes, none more than 2 cm in greatest dimension
pN2	Metastasis with a lymph node mass more than 2 cm but not more than 5 cm in greatest dimension; or more than 5 nodes positive, none more than 5 cm; or evidence or extra nodal extension of tumor
pN3	Metastasis with a lymph node mass more than 5 cm in greatest dimension
*Distant metastasis* (*M*)	
Mx	Distant metastasis cannot be assessed
M0	No distant metástasis
M1	Distant metastasis (M1a Non-regional lymph node or lung, M1b other sites)
*Serum tumors markers* (*S*)	
S0	Marker study levels within normal limits
S1	LDH < 1.5 × ULN and BHCG < 5000 mlu/ml and AFP < 1000 ng/ml
S2	LDH 1.5––10 × N or BHCG 5000–50,000 mlu/ml or AFP 1000–10,000 ng/ml
S3	LDH > 10 × ULN or BHCG > 50,000 mlu/ml or AFP > 10,000 ng/ml
*TNM stage*	
Stage IA	pT1 **N0** M0 S0
Stage IB	pT2-4 **N0** M0 S0
Stage IS	Any pT **N0** M0 S1-3
Stage IIA	Any pT **N1** M0 S0/S1
Stage IIB	Any pT **N2** M0 S0/S1
Stage IIC	Any pT **N3** M0 S0/S1
Stage IIIA	Any pT Any N **M1a S0/S1**
Stage IIIB	Any pT **N1-3 M0 S2,** Any pT Any N **M1a S2**
Stage IIIC	Any **pT N1-3** M0 **S3,** Any pT Any N **M1a S3,** Any pT Any N **M1b, Any S**

**Table 3:** Prognostic-based staging system for metastatic germ cell cancer according to the International Germ Cell Cancer Collaborative Group (IGCCCG) [7]

Group	Non-seminoma	Seminoma
Good prognosis	**All of the following criteria** Testis/retroperitoneal primary No non-pulmonary visceral metastases AFP < 1000 ng/ml BHCG < 5000 iu/l LDH < 1.5 × ULN	**All of the following criteria** Any primary site No non-pulmonary visceral metastases Normal AFP Any BHCG Any LDH
Intermediate prognosis	**All of the following criteria** Testis/retroperitoneal primary No non-pulmonary visceral metastases AFP 1000–10,000 ng/ml or BHCG 5000–50,000 ui/l or LDH 1.5–10 × ULN	**All of the following criteria** Any primary site Non-pulmonary metastases Normal AFP Any BHCG Any LDH
Poor prognosis	**Any of the following criteria** Mediastinal primary Non-pulmonary metastases AFP > 10,000 ng/ml or BHCG > 50,000 ui/l or LDH > 10 × ULN	**No patients classified as poor prognosis**

## Treatment of stage I seminoma

Approximately 75–80% of patients with seminoma present with stage I disease, with a survival rate of 99% that is independent of the chosen strategy. The most accepted treatment options are active surveillance and adjuvant chemotherapy with one course of single-agent carboplatin (AUC 7) [8, 9]. Carboplatin has demonstrated to be equally effective in reducing relapses compared with radiotherapy and is associated with less protracted toxicities [8] (I, A). The low incidence of relapses (15–20%, most in the retroperitoneum and within the first 14 months) and their high curability make surveillance a preferable option for patients compliant with follow-up.

Tumor size (either over 4 cm or as a continuous variable) and rete testis invasion are the most widely used (but not universally accepted) predictive factors for relapse on surveillance. The Spanish Germ Cell Cancer Group and the Swedish and Norwegian Testicular Cancer Group studies have validated them and support their utility to guide a risk-adapted, adjuvant chemotherapy approach [10, 11].

*Recommendation* Patients with stage I seminoma should be informed about the pros and the cons associated with each treatment approach, and the final decision would be balanced with the predicted risk of relapse and his individual desires and expectations. Active surveillance for most (willing and able) patients and short adjuvant chemotherapy for selected (high-risk or non-compliant) patients seem appropriate alternatives (III, B).

Relapses on surveillance or after adjuvant carboplatin can successfully be salvaged using the standard cisplatin-based chemotherapy adequate for their stage.

## Management of stage I non-seminoma

About two-thirds of patients with non-seminomatous testicular tumors are diagnosed with stage I disease. Orchiectomy alone cures approximately 75% of these patients. The rest of them will relapse, usually within the first 2 years after surgery, and the majority as good-risk advanced disease. The presence of lymphovascular invasion in the primary tumor defines a subgroup with high risk of relapse, approaching 50% in several series (in contrast to a 15% in the rest of patients) [9]. Five-year disease-specific survival of stage I non-seminoma patients is close to 100%, irrespective of the therapeutic alternative performed.

Two different approaches are available. Active surveillance for all patients provides an excellent cure rate and avoids unnecessary therapy and potential long-term toxicity in many patients [9, 12]. Alternatively, a risk-adapted approach, i.e., the administration of adjuvant chemotherapy for high-risk patients, allows a less intense follow-up, reduces the stress and life disruption associated with relapse, and decreases the need of postchemotherapy retroperitoneal lymphadenectomy. Two cycles of adjuvant BEP chemotherapy (Table 4) have been administered in the majority of studies [13]. However, some recent series suggest that a single cycle could be enough [14]. Retroperitoneal lymphadenectomy is being progressively abandoned as alternative for high-risk patients, because it appears to be less effective than adjuvant chemotherapy and constitutes overtreatment in many patients [15].

**Table 4:** Chemotherapy regimens in germ cell testicular cancer

BEP			
Cisplatin	20 mg/m^2^	Days 1–5	Repeat every 21 days
Etoposide	100 mg/m^2^	Days 1–5	
Bleomycin	30 mg	Days 1, 8 and 15	
EP			
Cisplatin	20 mg/m^2^	Days 1–5	Repeat every 21 days
Etoposide	100 mg/m^2^	Days 1–5	
VIP			
Cisplatin	20 mg/m^2^	Days 1–5	Repeat every 21 days
Etoposide	75 mg/m^2^	Days 1–5	
Ifosfamide	1.2 g/m^2^	Days 1–5	
Mesna	1.2 g/m^2^ CI	Days 1–5	
VeIP			
Cisplatin	20 mg/m^2^	Days 1–5	Repeat every 21 days
Vinblastine	0.11 mg/kg	Days 1 and 2	
Ifosfamide	1.2 g/m^2^	Days 1–5	
Mesna	1.2 g/m^2^ CI	Days 1–5	
TIP*			
Cisplatin	20 mg/m^2^	Days 1–5	Repeat every 21 days
Ifosfamide	1.2 g/m^2^	Days 1–5	
Mesna	1.2 g/m^2^ IC	Days 1–5	
Paclitaxel	250 mg/m^2^	Day 1	

* Several variations of this schedule exist

*Recommendation* In patients with stage I non-seminoma without lymphovascular invasion, active surveillance is recommended, with the exception of those cases in which poor compliance is expected. In patients with lymphovascular invasion, either surveillance or 1–2 cycles of adjuvant BEP are valid alternatives. Potential advantages and disadvantages of both approaches should be considered, considering individual patient preferences (III, B).

## Treatment of seminoma: advanced disease

Stage II-A (retroperitoneal lymph nodes 1–2 cm).

The classical treatment for patients with stage II-A seminoma has been radiotherapy. Despite the good disease control achieved with this approach, the risk of long-term, radio-induced neoplasms, and the excellent results of chemotherapy in this setting make this second option currently preferred for many clinicians.

*Recommendation* We recommend three cycles of BEP chemotherapy as the standard treatment if the patient does not have an increased risk of bleomycin-induced lung toxicity. Where this risk exists, four cycles of EP chemotherapy may be a good option [16–19] (III, B).

However, radiotherapy could also be an alternative in selected cases with risk of chemotherapy toxicity or patient preference. In these patients, radiotherapy to para-aortic and ipsilateral iliac lymph nodes with 30 Gy in 2 Gy fractions is recommended [20] (III, B).

Stage II-B, II-C, and III (IGCCCG low risk).

*Recommendation* Even though radiotherapy was the classic treatment for stage II-B patients, three cycles of BEP chemotherapy is today the recommended approach to prevent relapses, especially outside the radiation therapy field [16, 17] (II, B). For stages II-C and III considered as low risk as per the IGCCCG classification, three cycles of BEP chemotherapy are also the standard treatment [18] (I, A). Four cycles of EP chemotherapy are a reasonable option in cases with risk of bleomycin lung toxicity [19] (II, B).

Stage III (IGCCCG intermediate risk)

*Recommendation* Patients with intermediate risk (i.e., extrapulmonary visceral metastasis), stage III should be treated with four cycles of standard BEP chemotherapy. If there is any concern about an increased probability of bleomycin lung toxicity, this drug might be replaced by ifosfamide (i.e., four courses of VIP) [21] (I, A).

## Treatment of non-seminoma, advanced disease

Treatment recommendations for these patients are mainly based on the IGCCCG prognostic classification. As a general rule for all these patients, dose reductions should be avoided as treatment efficacy could be compromised; the routine use of prophylactic G-CSF from the first cycle is not clearly indicated, but it should be prescribed if severe neutropenia appears afterwards. It is also important to monitor weekly the decline in serum tumor markers after the first cycle, until they return to normal levels. It is recommended to perform serial diffusing capacity of the lung for carbon monoxide (DLCO) tests in patients who are planned to receive bleomycin. A DLCO drop over 30% and/or the onset of respiratory symptoms should prompt to omit this drug.

For patients with good-risk germ cell tumors, three cycles of BEP are considered standard therapy. In special situations, four cycles of EP can be an alternative option if patient comorbidities preclude the use of bleomycin [18, 19]. With these therapies, 5-year PFS and OS rates approach 90%.

For intermediate-risk patients, four cycles of BEP remain the standard choice, with a 5-year OS rate about 80%. If bleomycin cannot be used, VIP × 4 cycles could be an alternative option [21].

For poor-risk patients, four cycles of BEP are also the standard therapy, although the outcomes in this subgroup are poorer, with 5-year OS rates of 50%. In patients presenting with poor performance status, high tumor burden, or very extensive pulmonary or liver metastases, a first cycle of reduced-dose therapy (usually EP × 3 days) may decrease acute mortality without compromising long-term outcome. For poor-risk patients with slow marker decline, the role of intensification therapy remains controversial; the results of the GETUG-13 trial may indicate a benefit in PFS for patients with non-satisfactory marker decline after BEP first cycle with intensification, but it cannot be considered as a standard strategy [22]. It is strongly advised that patients with poor prognosis tumors should be referred to centers with experience managing these patients, and enrolled in clinical trials if available.

*Recommendation* For good-risk patients, BEP × 3 or EP × 4 are standard therapy (I, A).

For intermediate- and poor-risk patients, BEP × 4 is considered as standard therapy (I, A).

## Management of residual masses

Approximately 30% of metastatic GCTC will present residual masses (RM) after the first-line chemotherapy and their management is one of the most important keynotes for achieving high curability of these neoplasms [23].

1. After chemotherapy and normalization of serum tumor markers:
  - Non-seminoma:
    i. RM > 1 cm: There is a universal consensus to remove these masses completely. The extension of retroperitoneal surgery depends on the size and number of lymph-nodes affected. It is recommended to use templates if multiple involvement exists and bilateral lymphadenectomy in growing teratoma cases [24, 25]. In general, the pathologic findings after RM resection are viable carcinoma in 10–15%, teratoma in 35–40%, and necrosis-fibrosis in the rest 40–50% [24]. Nerve-sparing retroperitoneal lymph-node dissection (NS-RLND) should be performed at centers with extensive experience with this surgical procedure to avoid the impact on fertility and increasing the complete resection rate [23].
    ii. RM < 1 cm: The majority of authors and international guidelines recommend surveillance in patients achieving complete remission after chemotherapy [23, 24].
  - Seminoma:
    i. RM > 3 cm. There are special difficulties to perform radical surgery in RM from seminoma because of the frequent fibrotic reaction. A PET scan should be performed at least 8 weeks after finishing the last chemotherapy cycle. If the PET is unambiguously positive or the RM is clearly growing, a resection of the mass should be performed [26].
    ii. RM < 3 cm. Surveillance with imaging test is recommended with no need of surgery [23, 26].
2. Salvage surgery in refractory disease (desperation surgery). Some cases with oligo-metastatic disease resistant to various lines of chemotherapy and elevated AFP can be rescued with surgery. Long-time survival may be obtained in 30–70% of completely resected procedures [27, 28].

As a general rule, RM greater than 1 cm should be resected from all locations. The presence of fibrosis in a retroperitoneal residual mass does not exclude the presence of teratoma in thoracic metastases (up to 20% of cases). Only patients with necrosis in both retroperitoneum and in one side of the lung can avoid contralateral lung resections [29].

*Recommendation* Complete resection of RM should be performed after chemotherapy in: All RM > 1 cm in non-seminoma cases (III, A). Only RM > 3 cm in seminoma patients with unequivocally positive PET scan (III, B). Chemo-refractory patients with no raised markers or only elevation of AFP (IV, B). Uni or bilateral NS-RLND or template procedures should be performed by expert hands at reference centers (III, B).

## Salvage therapy

Patients that relapse or progress after the first-line chemotherapy comprise a heterogeneous group of patients with varied outcomes, and a 2 year survival rate that ranges from 6 to 75% according to the International Prognostic Factor Study Group (IPFSG) risk classification (Table 5) [30].

**Table 5:** International Prognostic Factor Study Group risk classification [30]

	Score points			
	0	1	2	3
Primary	Gonadal	Extragonadal		Mediastinal non-seminoma
Prior response	CR/PRm−	PRm+/SD	PD	
PFI	>3 months	≤3 months		
AFP	Normal	≤1000	>1000	
BHCG	≤1000	>1000		
LBB	No	Yes		

*Histology score* seminoma = −1; non-seminoma or mixed = 0

*Risk groups* (*sum of scores*) very low = −1; low = 0; intermediate = 1; high risk = 2; very high risk = 3

*CR* complete response; *PRm−* partial response markers negative; *PRm+* partial response markers positive; *SD* stable disease; *PD* progression disease; *PFI* platinum-free interval; *AFP* alpha-fetoprotein at salvage treatment; *BHCG* human chorionic gonadotrophin at salvage treatment; *LBB* liver, bone or brain metastasis

Salvage treatment is based on chemotherapy and salvage surgery. When possible, it is recommended to refer these patients to experienced high-volume centers. Conventional-dose chemotherapy (CDCT) includes regimens that contain cisplatin and ifosfamide plus either etoposide (VIP), vinblastine (VeIP), or paclitaxel (TIP) are effective mainly in patients with good-risk features as those with primary gonadal tumors and good response to the previous first-line chemotherapy [31]. However, more than a half of these patients will progress from CDCT.

High-dose chemotherapy (HDCT) has demonstrated to cure a significant proportion of patients who relapse after CDCT and should be considered as a reasonable option for patients fit enough for this treatment. Phase III clinical trials including HDCT as the first-line salvage therapy are scarce and inconclusive. A large international retrospective pooled-data analysis [32] has suggested a benefit for HDCT over CDCT as the first-line salvage therapy, with a 35% reduction in the risk of death. This encouraging study prompted the development of an intergroup prospective randomized trial comparing HDCT to CDCT in the first salvage treatment (the TIGER study). This kind of trial is the best option for GCTC patients that fail first-line chemotherapy. When a clinical trial is not available, treatment should be based on the patient characteristics and medical experience [33]. Surgery is mandatory after salvage chemotherapy if there is any RM according to the previously described criteria and should be performed as soon as the patient is fit for the intervention.

*Recommendation* Patients who experience failure on the first-line cisplatin-based chemotherapy should be referred to (or at least consult with) a center with experience in GCTC salvage treatment (IIIA). HDCT cures selected patients who experience disease progression on CDCT rescue regimens and should be offered to patients fit enough for HDCT (IIIA). A clinical trial comparing HDCT and CDCT is the preferred option for patients who experience failure on the first-line cisplatin-based chemotherapy (IIIA). Surgery to remove residual masses is encouraged whenever possible after salvage treatment (IIIA).

## Toxicity and late effects in long-term survivors

GCTC is one of the few tumors in which more than 90% of metastatic patients are cured. Therefore, understanding the long-term effects of therapy and developing research studies and guidelines in this field are important to optimize the care in this population. Fertility, cardiovascular toxicity, specific sequela from chemotherapy, incidence of second neoplasms, and other chronic health-related issues are the main long-term toxicities to be discussed.

### Fertility

Pre-existing fertility problems may be aggravated by chemotherapy in patients with GCTC. Fatherhood rate among testicular cancer survivors wishing to father a child is around 70%. No increased risk of malformations is found in children of GCC survivors [34].

*Recommendation* Patients should be informed about fertility odds. Cryopreservation should be offered to all patients before chemotherapy.

### Cardiovascular toxicity

Chemotherapy-related cardiovascular toxicity is a result of both direct endothelial damage induced by cisplatin and indirect hormonal and metabolic changes. Metabolic syndrome affects as much as 30% of long-term GCTC survivors [35, 36]. One of the potential factors associated to the early metabolic syndrome is male hypogonadism, observed between 11 and 35% among GCTC survivors [37].

*Recommendation* Long-term survivors should receive appropriate counselling about cardiovascular health. Monitoring of blood pressure, cholesterol and status of tobacco consumption, and physical activity should be stated. Determination of testosterone during follow-up and substitutive therapy should be used as needed.

It is mandatory to minimize the amount of therapy received per patient.

### Specific sequela of chemotherapy

Chronic neurotoxicity occurs in half of men, whereas severe hearing loss is as high as 20% [38]. Some degree of renal function impairment occurs in up to 30% [39]. Pulmonary fibrosis, occurring in 5–10% of patients treated with bleomycin, is fatal in 1%. Most of these toxicities are dose-related, again emphasizing the importance to limit therapy as much as possible.

*Recommendation* Adequate evaluation of hearing loss and appropriate counselling about preservation of renal function are recommended.

Patients and physicians should be advised to avoid further toxic interventions.

### Second neoplasms

The relative risk of a second solid non-germ-cell tumor is approximately doubled after radiotherapy or chemotherapy. These figures are particularly high for malignancies of the gastrointestinal and urinary tracts. Secondary solid tumors usually occur ≥10 years after treatment as opposed to chemotherapy-induced leukaemia which emerge within one decade after treatment. The estimated cumulative risk of leukaemia is 0.5 and 2% after cumulative etoposide doses of <2 and >2 g/m^2^, respectively.

### Other health-related issues

Health-related quality of life (HRQoL) in long-term GCTC survivors seems to be similar to the normal male population, but persisting long-term treatment-related side-effects show a strong association with both impaired physical and mental HRQoL. The level of anxiety is higher in GCTC survivors than in the general male population. The prevalence of self-reported chronic fatigue is common among patients with GCTC [23].

*Recommendation* Proper psychological evaluation should be available for long-term survivors of germ cell cancer.

## Extragonadal germ cell tumors

Approximately 2–5% of germ cell tumors are of extragonadal origin. The most common sites are in the midline: mediastinum (PM-EGCT) and retroperitoneum, followed by the central nervous system (CNS) [40, 41]. They are classified with the same IGCCCG criteria [7]. The prognosis of extragonadal seminomas is favorable regardless of its location, whereas that of non-seminomatous tumors (NS-EGCT) is indisputably unfavorable [40–42]. PM-EGCT appears to represent a clinically and biologically distinct disease entity, associated with lower complete response rates, high rates of relapse, and frequent failure to salvage chemotherapy. Presence of visceral disease (liver, lung, or CNS metastases), a primary mediastinal location site, and elevation of BHCG are independent prognostic factors for survival in NS-EGCT [42]. LDH and AFP are less predictive for survival. The main prognostic factor related to refractoriness to therapy is non-seminomatous histology. The management of primary CNS germ cell cancer is complex, requires the integration of neurosurgeons, radiation oncologist, and medical oncologists, and should be performed in experienced institutions.

*Recommendation* Extragonadal germ cell tumors should be treated with cisplatin-based combinations (III, A). Four cycles are recommended for poor-risk (mediastinal) patients, although treatment in a clinical trial is preferred (IV, A). Surgical resection is mandatory for patients with any visible residual mass and serum tumor marker normalization following the first-line chemotherapy (IV, A). Primary CNS germ cell cancer should be referred to experienced institutions.

## Late relapse

Late relapse of GCTC is uncommon: 1.4% in seminomas and 3.2% in non-seminomas. It is defined as recurrence occurring >2 years after the completion of the primary treatment for metastatic testicular cancer (with at least three cycles of chemotherapy). This definition excludes patients who relapse after adjuvant treatment or during surveillance who are usually cured by chemotherapy alone [23]. Patients with late relapse represent a subgroup with an adverse prognosis, do not tend to respond favorably to new chemotherapy, are usually AFP-positive, and often contain mature teratoma and/or non-GCTC elements [28, 43]. If technically feasible, all lesions should be removed by radical surgery at experienced centers. Further chemotherapy must be individualized according to histology and serum tumor markers. If salvage chemotherapy is the first treatment option for a late relapse (particularly for patients with rapidly raising BHCG), surgery should be conducted whenever possible.

*Recommendation* Late relapses are uncommon and must be considered initially for radical surgery rather than chemotherapy, as those tumors tend to be chemo-resistant (IV, A).

## Brain metastases

Brain metastases (BM) can be present at the initial diagnosis or at relapse [44, 45]. Their prognosis is poor. Adverse risk factors for both groups are: (1) the multiplicity of BM; and (2) the presence of liver or bone metastases. For patients with synchronous BM, chemotherapy is the standard of care with BEP × 4. These patients have better prognosis. Radiotherapy and/or neurosurgery should be offered to patients with adverse risk factors (i.e., primary mediastinal non-seminoma). For patients with metachronous BM, chemotherapy has worse results, suggesting that chemotherapy resistance is critically related to outcome. The use of HDCT and multimodality treatment (radiotherapy/neurosurgery) are associated with a better outcome, especially in patients with poor prognostic factors (AFP > 100 ng/ml or BHCG > 5000 U/L) [45]. If clinically indicated and feasible, surgical resection of the metastasis should also be performed.

*Recommendation* Chemotherapy should remain the standard of care in patients with BM at the initial diagnosis (IV, A). Additional radiation therapy and/or neurosurgery may be individualized (IV, B). The use of HDCT and local treatment may improve the outcome for patients who experience relapse with metachronous BM (IV, B).

## Follow-up

The early detection and treatment of relapse represent the primary objective of follow-up visits during the first 5 years. Follow-up schedules are empirical and have never been validated. Table 6 summarizes current recommendations from the SGCCG in our country.

**Table 6:** Recommended follow-up schedules

Tumor	Stage	Treatment	Exploration	1st year (months)	2nd year (months)	3rd–5th year (months)
Seminoma	I	Surveillance	Markers*	4	6	6
			Chest X-ray	4	6	6
			Abdominal CT	4	6	12
Seminoma	I	Adjuvant carboplatin	Markers*	6	6	12
			Chest X-ray	6	6	12
			Abdominal CT	6	12	12
Non-seminoma	I	Surveillance	Markers	2	3	6
			Chest X-ray	4	6	6
			Abdominal CT	4	6	12
Non-seminoma	I	Adjuvant BEP	Markers	3	3	6
			Chest X-ray	6	12	12
			Abdominal CT	6	12	12
Seminoma Non-seminoma	II–III	Post BEP ± residual mass surgery	Markers*	3	3	6
			Chest X-ray	6	6	12
			Abdominal CT	6	6	12

* The use of serum tumor markers to guide or monitor treatment for advanced seminoma or to detect relapse in those treated for stage I seminoma is debatable
